# Validation to Spanish of the Caring Assessment Tool (CAT-V)

**DOI:** 10.1590/1518-8345.0920.2965

**Published:** 2017-10-19

**Authors:** Rosa María Fernández Ayuso, Juan Manuel Morillo Velázquez, David Fernández Ayuso, Julio César de la Torre-Montero

**Affiliations:** 1PhD, Professor, San Juan de Dios University School of Nursing and Physical Therapy, Universidad Pontificia Comillas, Madrid, Spain.; 2 PhD, Associate Professor, San Juan de Dios University School of Nursing and Physical Therapy, Universidad Pontificia Comillas, Madrid, Spain.; 3 PhD, Adjunct Professor, San Juan de Dios University School of Nursing and Physical Therapy, Universidad Pontificia Comillas, Madrid, Spain

**Keywords:** Behavior, Nursing Care, Validation Studies

## Abstract

**Objective:**

to translate and validate to Spanish the Caring Assessment Scale tool, CAT-V, by Joanne Duffy, within the framework of Jean Watson; as a secondary objective, it is proposed to evaluate its psychometric properties. There are tools designed to measure the patient’s perception of provided cares, including CAT-V, the subject of our interest, in a way that it can be used in Spanish-speaking patients.

**Methods:**

to meet the objectives, it was performed sequential translation and retro-translation of the scale to be validated, through a standardized procedure. The final version of that scale was validated in a sample of 349 patients from four public and two private hospitals in Madrid, Spain.

**Results:**

The instrument was translated and validated with high internal consistency (Cronbach’s alpha .953). The subsequent factor analysis revealed a three-factor structure, not coincident with the data from the US population.

**Conclusion:**

it is considered that the translation of CAT-V is a suitable instrument to be used in the evaluation of patient care in Ibero-american health centers whose language is Spanish.

## Introduction

Societies nowadays poses several strong demands in terms of efficiency and effectiveness, presented to health professionals working for them. The concept of patient and client is well established in both the public and private health, and this is only acceptable if this concept does not go against the perspective of quality of care and the humanization of care. The concept of quality has likewise been transformed in the last decades. If at a given moment it was focused on the techniques, procedures and scientific advances, now it also includes parameters such as the point of view of professionals, social impact of healthcare and to a greater extent, as an indicator of quality performance, and the valuation made by the patient and his family of the services received^1^.

The term “caring behaviors” was defined as what the nurses say or do when they transmit care to patients^2^. Some authors claim that those are actions typical of concern for the welfare of a patient, such as sensitivity, hospitality, attentive listening, honesty and nonjudgmental acceptance^3^. 

The concept of quality of care has been transformed in recent decades, departing from the techniques and procedures. It has also changed the valuation of other parameters, such as the point of view of professionals, the social impact implicit in healthcare delivery and the assessment the patients and their families of the services received, as well as conceptual changes regarding satisfaction, caused by the cultural change in health organizations^4^ ^-^ ^5^.

Satisfaction itself is multi-dimensional and complicated in its measurement, and also a changing and evolving complex concept^6^. As such, it depends on many factors, and only by putting the patient at the center of the care processes, is unquestionable that satisfaction can and should be measured in order to assess the care received. 

In order to evaluate and measure the behaviors of nursing care, numerous tools were developed, most of them in English-speaking countries. 

The first tool developed for this purpose was a form, the Care Satisfaction Questionnaire, CARE-Q, designed to assess the perceptions of patients and nurses about the importance of behavior in care^7^. It is a tool of 50 items arranged in six dimensions: accessible, explains and facilitates, accommodating, trusting relationship, anticipates needs, monitors and tracking.

For the elderly, the Caring Behaviors Inventory instrument of 28 items was developed. Although easier to use than the CARE-Q, it is limited in terms of the population that can be subject to that assessment^8^. After an evaluation of aspects of other instruments already used previously in research and applicable in the clinical settings, we considered the CAT-V questionnaire can be an ideal tool to evaluate the perception of the caring behaviors in the Spanish population, given its object of study, which is not so much the overall patient satisfaction with care, but focuses on the human aspects of such assistance provided by the nursing staff. The assessment of behavior is based on a theoretical model widely supported, as is the Theory of Human Care^9^ ^-^ ^10^. It is an easy applicable tool with patients, with an average length, and easily understandable short items. Internationally there have been several studies that define nursing care as an interactive and inter-subjective process that occurs in moments of shared vulnerability between the nurses and the patients. They are intended to provide patients’ comfort and this only occurs when nurses respond to patients in a care situation^8^ ^,^ ^11^ ^-^ ^14^. Studies were conducted in Spain to research the patients’ perceptions regarding nursing professionals from an ethical point of view^15^. The results showed the importance of personal relationships to patients, they expressed their perceived satisfaction when they were treated by nurses as human beings in all their dimension and felt they respected their privacy, also adding that nurses passed them safety and confidence. Most patients expressed the desire to receive information on the development of their disease and future expectations. Other authors conducted a study with the aim of knowing the perception of patients about what they consider important in relation to health care^16^. They concluded that patients perceived a wellbeing feeling if they had been well treated, in spite that they considered the technical aspects as important. The Caring Assessment Tool (CAT) was developed to assess the perception that patients have on nursing care behaviors^17^ ^-^ ^19^. Originated from the Theory of Human Care^12^, several items that correspond with each “care factor” were designed. However, no deep evaluation of psychometric properties was performed. Subsequently, the number of items was reduced in order to make it more feasible to use in care settings, and was presented in its CAT-IV version, consisting of 36 items. To perform validation and explore its psychometric properties, five hospitals in the United States were selected. Its target population consisted of inpatients with at least two days in hospital, ensuring that there had been enough interaction with the nursing staff. This instrument had an internal consistency of 0.96, and an internal structure of eight factors that allowed grouping items under new dimensions provided with a theoretical basis. However, in a subsequent study with a larger sample size and the participation of 12 hospitals in four different areas of the United States, with greater heterogeneity of patients, this internal structure was not kept stable, since the model with best fit was the instrument with a single factor^20^. 

This latter study allowed to check the burden presented to care staff by the administration and collection of the questionnaire CAT-IV, although there were benefits perceived by nurses e.g. how these professionals learned to recognize and educate themselves on the caring behaviors, taking the patients into consideration. The fact that 36 items still made the instrument too long, led to propose a reduction, discarding those items that have saturations of at least 0.70 and item-total correlations of at least 0.70. Thus, the resulting new version of 27 items has been called CAT-V, whose reliability (Cronbach’s alpha, 0.967) and internal structure of a single factor has been proven. This is the instrument that will be studied in the present research.

## Method

Design: An observational validation study of psychometric nature with translation and trans-cultural adaptation of an evaluation scale of care.

This research has been approved by the Ethics Committee of Research of the University Rey Juan Carlos, Madrid, Spain, as observational, psychological or behavioral research in humans. It also requested the authorization of the Clinical Research Ethics Committee of the University Hospital Ramon y Cajal in Madrid. In addition, authorization from the author of the scale was requested in order to carry out this investigation.

**Figure 1:**
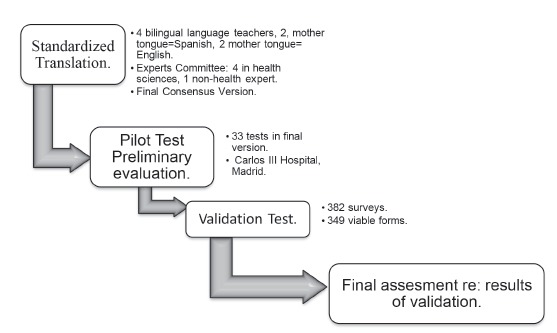
Flowchart of work process in the project

The objectives of this study, as can be seen in Figure 1, include: cross-culturally translating and adapting from English to Spanish the CAT-V rating scale for nursing care. Assessing the psychometric properties of the CAT-V scale translated for Spanish-speaking population and validating the CAT-V rating scale for nursing care for its use in Spanish-speaking population.

We can state the hypothesis of this paper as: the CAT-V scale translated will meet internal consistency characteristics suitable for its use in Spanish-speaking population, and will present a uni-dimensional structure similar to the original in English.

To carry out the process of standardized translation and adaptation of this research, we have followed recommendations of several authors with extensive experience in this field, following guidelines referring to direct and reverse translation of the tool^21^. Two professionals, both in the field of language teaching with Spanish as their mother tongue, and deep knowledge of English, one of them related to health sciences, and one without deep knowledge of health, were provided with the original scale to carry out, independently, the translation into Spanish. They were provided with a template to point out the difficulties or ambiguities that could be found in the translation of a particular item. They were asked to prioritize the equivalence of the concepts with the original English version, and not a literal translation. For the reverse translation of the tool we relied on the collaboration of two professionals with American English as their mother tongue and with identical characteristics as the previous experts. Once these translations were completed, a joint meeting with all the translation team was convened to conduct a detailed study of each item translated and check differences of opinion, so that could be reached a consensus version. We contacted the author of the original version of the scale in order to make an assessment of the reverse translation and look for significant differences between it and the meaning of each item in the original version. Thus, it was confirmed that the validity of the meaning of the instrument was maintained. Additionally we convened an expert committee consisting of five people, mainly professionals Nursing and other Health Sciences, and a non-member of the health sector. They received the Spanish version of consensus, in order to check if the understanding of each item was adequate, or whether it was necessary to improve the translation to suit the Spanish cultural environment. The result led to the final Spanish version of the CAT-V scale. Inclusion criteria in the study were: patients over 18 years old, conscious and oriented, capable of fully understanding spoken and written Spanish, and hospitalized in medical-surgical units for at least two nights.

The target population was first approached through an initial assessment of understanding of the tool in a group of 33 patients of the Hospital Carlos III. After this phase, no changes were needed in the final version of the questionnaire. Subsequently, a total of 382 surveys were collected in different public hospitals in Madrid, Spain: Hospital Carlos III, Getafe University Hospital, University Hospital Ramon y Cajal, University Hospital October 12^th^ , and two private hospitals belonging to the Quirón Group: Hospital Quirón Madrid and Hospital Quirón Vizcaya. 

Given that the scale has 27 items, and that the recommendations regarding the sample size indicate that there is need of a ratio of 10 subjects per item in order to be able to carry out an exploratory factor analysis, we planned to have a minimum of 270 subjects^22^ ^-^ ^24^. The collected data were entered into a database and analyzed using SPSS version 19.0 for Windows. The item analysis was performed by calculating the value of the 25th and 75th percentiles of the sample according to the overall score of the CAT-V scale, and then obtaining the mean and standard deviation for each item in these percentiles. Finally we confirmed that there were statistically significant differences between these percentiles by applying the Student *t* test for independent samples, establishing a significance level of p <0.05. For the analysis of internal consistency, Cronbach’s alpha coefficient and item-total correlation was calculated. Finally, the structure of factors was analyzed by an exploratory factor analysis with Varimax rotation.

## Results

Two well-defined phases of this project are thus outlined: the standardized translation of the Care Assessment scale and then the validation phase of this scale. 

The process of standardized translation reached a full consensus of the four translators and expert panel on the right approach of the items, after performing the appropriate steps in this type of translation, following the methodology described and supported by the literature. The results of the standardized translation process have been unanimous, conclusive and confluent in a complete layout of 27 items in Spanish. During the validation study, a total of 382 patients were surveyed. In order to minimize the loss of information due to incomplete data from some surveys, we proceeded to clean the database, which resulted in a final base consisting of 349 patients/forms. 

The average age was 57.36 years with a standard deviation of 16,869. The age range was 18 to 93 years. Regarding gender distribution, there was parity in the sample. Regarding the score scale CAT-V, the possible range is between 27 and 135. In the sample, the range was between 37 and 135, with an average of 115.24 points and a standard deviation of 16,208. The best-valued item was number 11 (They respect me) with an average score of 4,816, and the worst score was item number 17 (They help me to formulate questions to other health providers), with an average score of 3,375. 

For item analysis and reliability of the scale, a study of the items by percentiles was performed. For this purpose, it was conducted a calculation of CAT-V scores corresponding to the 25th percentile, which was 106.73; and to the 75th percentile, which was 127. Based on these values, descriptive statistics of both subgroups of the sample for each item were calculated. Subsequently, a Student t test for independent samples was applied in order to compare both subgroups (25th and 75th percentile of the total score) for each item. The difference was statistically significant (p <0.001) between the means of both subgroups in each of the items, supporting the suitability of them to continue the study of the properties of the instrument. As shown in Table 1, it was calculated Cronbach’s alpha coefficient as an indicator of the internal consistency of the instrument. The value obtained was 0.953, indicating high reliability. Finally, a study of the item-total correlation was performed. All items rated above 0,500, with the exception of Item 3 (They respect my beliefs), with a correlation coefficient of 0.386. However, should we opted for the removal of this item, Cronbach’s alpha would grow only one thousandth (up to 0.954). Conversely, the deletion of any of the other items would maintain or reduce reliability. For factor analysis, the first step was to calculate the measure of sampling adequacy Kaiser-Mayer-Olkin (KMO) test and Bartlett’s sphericity in order to confirm if it was suitable to perform an exploratory factor analysis from the sample studied. KMO value (0.949) and significance of Bartlett test (p <0.001) supported the implementation of the factorial analysis.

**Table 1:** Item-total correlation and Cronbach’s α in Caring Assessment Scale. Madrid, Spain, 2013

Items of Caring Assessment scale CAT-V*	**Item-total correlation**	**Cronbach’s α**
**1. They help believe in myself.**	**0,610**	**0,952**
**2. They make feel as comfortable as possible.**	**0,585**	**0,952**
**3. They respect my beliefs.**	**0,386**	**0,954**
**4. They pay attention when I speak.**	**0,609**	**0,952**
**5. They help me see a positive side in my situation.**	**0,697**	**0,951**
**6. They help me feel less concern.**	**0,711**	**0,951**
**7. They anticipate my needs.**	**0,662**	**0,951**
**8. They let me choose the best moment to talk about my concerns.**	**0,699**	**0,951**
**9. They care about my point of view.**	**0.724**	**0,951**
**10. They show interest in me.**	**0,722**	**0,951**
**11. They respect me.**	**0,567**	**0,953**
**12. They answer to my family with sensitivity.**	**0,647**	**0,952**
**13. They acknowledge my feelings.**	**0,626**	**0,952**
**14. They help me to clarify what I think about my illness.**	**0,725**	**0,951**
**15. They help me find different approaches to my illness troubles.**	**0,772**	**0,950**
**16. They ask me what do I know about my illness.**	**0,619**	**0,953**
**17. They help me to formulate questions to other health providers.**	**0,601**	**0,953**
**18. They foster my hope.**	**0,717**	**0,951**
**19. They respect my need for privacy.**	**0,556**	**0,952**
**20. They ask my opinion about how my illness is going.**	**0,663**	**0,952**
**21. They handle my body carefully.**	**0,502**	**0,953**
**22. They help me with my sleeping routines.**	**0,638**	**0,952**
**23. They encourage my attitude to move on.**	**0,742**	**0,951**
**24. They help me to deal with negative feelings.**	**0,757**	**0,950**
**25. They know what is important for me.**	**0,727**	**0,951**
**26. They speak openly with my family.**	**0,648**	**0,951**
**27. They show respect for everything that is important for me.**	**0,698**	**0,951**

*CAT-V: Caring Assessment Tool.

Regarding the commonalities of each item, after applying principal component analysis as extraction method, all items except four, rated above 0.5 and are represented equally in the factor analysis. Items with lower saturation were 3 (They respect my beliefs), 19 (They respect my need for privacy), 21 (They handle my body carefully) and 22 (They help me with my sleeping routines). Regarding the variance, a three factors structure explained 59.327% of the total, but actually one of the factors predominates with 47.387% and the other two factors explained 7.194% and 4.746% respectively.

## Discussion 

There were not validated instruments available in Spain, capable to measure these aspects and able to be applied systematically to hospitalized patients. While at first we thought in designing an original scale, it was considered that it could be more operational and useful regarding possible comparisons with other studies from other countries, we then opted for the process of translation and adaptation of an instrument that has been previously validated, and whose psychometric properties have been analyzed, such as CAT-V, into Spanish, applicable in Spain and Latin America. 

There is a previous version in US-Latino Spanish, which has not been previously validated^20^. For this reason, we started with the original English version to carry out the process of cultural adaptation to the Spanish population. The results of the application for validation indicate that it presents high reliability, and an internal structure constituted by three factors, one of which has a clear predominance over the other two. It is considered that the main contribution of this work is the internal structure of three factors as mentioned in the results. This structure clearly contrasts with the results obtained with the original scale by the author of the instrument. In her study, a uni-factorial structure of the instrument was found. Each of the extracted factors of our analysis showed good internal consistency, suggesting that may represent different dimensions of care. One last important aspect is that a confirmatory factor analysis was not performed using the conclusions drawn from exploratory analysis. We considered that for such analysis we would have required a different starting sample and more participants to validate the results of the confirmatory analysis. It is important to note the evolution in the administration of such questionnaires, including the electronic forms^24^, with data analysis in real time. The current trend, patient-centered care^25^ indicates that these tools are useful for both patients and professionals who want to develop specific questionnaires on the needs of patients about themselves in different settings and people in different situations^26^ ^-^ ^28^. Finally, it is crucial to stress the importance of assessing care, to measure its quality from the point of view of the patient, within the vision of personalized nursing, where the patient is the protagonist of the process^29^. Knowing the basics of care, help us to provide better health service^30^.

## Conclusion

The objective of this research has been completed, as we have translated and psychometrically analyzed the Spanish version of the scale Caring Assessment Tool (CAT-V). The final version of the questionnaire Care Assessment CAT-V in Spanish has a layout consisting in 27 items, with the possibility to be answered in a Likert scale with 5 points (never, rarely, sometimes, often, always). The findings of this study meet the proposed objectives: 1. - It has been successfully performed the translation into Spanish of the Caring Assessment Tool (CAT-V), respecting the structure of 27 items from the original version in English. 2. The final version validated in Spanish CAT-V, has demonstrated high internal consistency in the Spanish-speaking sample population studied. 3. The psychometric study reveals a different dimensional structure, which does not appear in the original version. This study provides a tool for assessing patients’ perception of care, presenting a subjective constraint. To have an instrument of this type opens the door in order to allow the health care settings to reflect on the necessary humanization of care we give to patients. Also this tool is useful for teaching, so that future nursing professionals may know the patients’ understanding about what is to be cared for; and from the beginning of its training the humanizing component of its work appears as of fundamental importance in their curriculum. It is our mission to be responsive to the needs of patients meeting their demands based on the standards of the required professional performance, maintaining a high level of competence in all areas of care.

## References

[1] Mira JJ, Aranaz J (2000). La satisfacción del paciente como una medida del resultado de la
atención sanitaria. Med Clin.

[2] Cronin SN, Harrison B (1988). Importance of nurse caring behaviors as perceived by patients after
myocardial infarction. Heart Lung.

[3] Modic MB, Siedlecki SL, Quinn- Griffin MT, Fitzpatrick JJ (2014). Caring behaviors: Perceptions of acute care nurses and hospitalized
patients with diabetes. J Patient Experience.

[4] Mira J, Lorenzo S, Rodríguez-Marín J, Aranaz J, Sitges E (1998). La aplicación del modelo europeo de gestión de la calidad total
alsector sanitario: ventajas y limitaciones. Rev Calidad Asistencial.

[5] Mira J (1992). La satisfacción del paciente. Aspectos teóricos y
metodológicos. Rev Psicol Sal.

[6] Barrasa JI, Aibar C (2003). Revisión sistemática de los estudios de satisfacción realizados en
España en el período 1986-2001. Rev Calidad Asistencial.

[7] Zamanzadeh V, Azimzadeh R, Rahmani A, Valizadeh L (2010). Oncology patients’ and professional nurses’ perceptions of important nurse
caring behaviors.

[8] Wolf ZR (2012). Nursing practice breakdowns: good and bad nursing. Med Surg Nurs.

[9] Watson J, Brewer BB (2015). Caring science research: criteria, evidence, and
measurement. J Nurs Adm.

[10] Cara C, O’Reilly L, Kérouac S (2004). A better humanization of care: real possibility or
utopia. Perspect Infirm.

[11] Gaut D (1983). Development of a theoretically adequate description of
caring. Western Journal of Nurs Res.

[12] Watson J (1979). Nursing: The Philosophy and Science of Caring.

[13] Wolf ZR (1986). The caring concept and nurse identified caring
behaviors. Top Clin Nurs.

[14] Wolf ZR, Colahan M, Costello A, Warwick F, Ambrose MS, Riviello E (1998). Relationship Between Nurse Caring and Patient
Satisfaction. Med Surg Nurs.

[15] Rojo M, Sáenz de Buruaga M, Rueda MJ, Sola MT, Fernández ML (2000). Las actitudes éticas del cuidado desde la percepción de los pacientes
mayores, en diálisis. Enferm Nefrol.

[16] Moreno Monsiváis MG, Interial MG, Ruiz P, Almansa MP (2012). Percepción del paciente acerca de su bienestar durante la
hospitalización. Index Enferm.

[17] Larson PJ (1995). Important nurse caring behaviors perceived by patients with
cáncer.1984. Oncol Nurs Forum.

[18] Duffy JR, Brewer BB (2011). Feasibility of a multi-institution collaborative to improve
patient-nurse relationship quality. J Nurs Adm.

[19] Duffy JR, Hoskins L, Seifert RF (2007). Dimensions of caring: psychometric evaluation of the caring assessment
tool. Adv Nurs Sci.

[20] Duffy JR, Brewer BB, Weaver MT (2014). Revision and psychometric properties of the Caring Assessment
Tool. Clin Nurs Res.

[21] Gjersing L, Caplehorn J, Clausen T (2010). Cross-cultural adaptation of research instruments: language, setting,
time and statistical considerations. Med Res Met.

[22] Morales Vallejo P (2013). Análisis factorial en la construcción e interpretación de tests, escalas y
cuestionarios.

[23] Sousa V, Rojjanasrirat W (2011). Translation, adaptation and validation of instruments or scales for
use in cross-cultural health care research: a clear and user-friendly
guideline. J Eval Clin Pract.

[24] Duffy JR, Koolen WC, Wolverton CL (2012). Evaluating Patient-centered Care: Pilot Study Testing Feasibility of
Electronic Data Collection in Hospitalized Older Adults. J Nurs Care Qual.

[25] Flagg AJ (2015). The role of patient-centered care in nursing. Nurs Clin North Am.

[26] Van Vliet L, Harding R, Bausewein C, Payne S, Higginson I (2015). How should we manage information needs, family anxiety, depression,
and breathlessness for those affected by advanced disease: development of a
clinical Decision Support Tool using a Delphi design. BMC Med.

[27] While AE, Clark L. (2014). Development of a competency tool for adult trained nurses caring for
people with intellectual disabilities. J Nurs Manag.

[28] AT J, Amit Dias A, Philp I, Beard J, Patel V, Prince M (2015). Identifying common impairments in frail and dependent older people:
validation of the COPE assessment for non-specialised health workers in low
resource primary health care settings. BMC Geriatr.

[29] (2015). The importance of nurses in cancer cares. Lancet Oncol.

[30] Santos MR, Bousso RS, Vendramim P, Baliza MF, Misko MD, Silva L (2014). The practice of nurses caring for families of pediatric inpatients in
light of Jean Watson. Rev Esc Enferm USP.

